# Smoking Is Associated with, but Does Not Cause, Depressed Mood in Pregnancy – A Mendelian Randomization Study

**DOI:** 10.1371/journal.pone.0021689

**Published:** 2011-07-19

**Authors:** Sarah J. Lewis, Ricardo Araya, George Davey Smith, Rachel Freathy, David Gunnell, Tom Palmer, Marcus Munafò

**Affiliations:** 1 School of Social and Community Medicine, University of Bristol, Bristol, United Kingdom; 2 MRC Centre for Causal Analyses in Translational Epidemiology (CAiTE), School of Social and Community Medicine, University of Bristol, Bristol, United Kingdom; 3 Genetics of Complex Traits, Institute of Biomedical and Clinical Sciences, Peninsula College of Medicine and Dentistry, University of Exeter, Exeter, United Kingdom; 4 School of Experimental Psychology, University of Bristol, Bristol, United Kingdom; University of Ottawa, Canada

## Abstract

Smokers have a higher prevalence of major depressive episodes and depressive symptoms than the general population, but whether this association is causal, or is due to confounding or reverse causation is uncertain because of the problems inherent in some epidemiological studies. Mendelian randomization, in which a genetic variant is used as a surrogate for measuring exposure, is an approach which may be used to better understand this association. We investigated the rs1051730 single nucleotide polymorphism in the nicotine acetylcholine receptor gene cluster (*CHRNA5-CHRNA3-CHRNB4*), associated with smoking phenotypes, to determine whether women who continued to smoke were also more likely to report a low mood during pregnancy. We found among women who smoked pre-pregnancy, those with the 1051730 T allele smoked more and were less likely to quit smoking during pregnancy, but were also less likely to report high levels of depressed mood at 18 weeks of pregnancy (per allele OR = 0.84, 95%CI 0.72 to 0.99, p = 0.034). The association between genotype and depressed mood was limited to women who were smokers prior to pregnancy, with weak evidence of an interaction between smoking status and genotype (p = 0.07). Our results do not support a causal role of smoking on depressed mood, but are consistent with a self-medication hypothesis, whereby smoking is used to alleviate symptoms of depression. A replication study using multiple genetic variants which influence smoking via different pathways is required to confirm these findings and provide evidence that the genetic variant is reflecting the effect of quitting smoking on depressed mood, and is not directly affecting mood.

## Introduction

Cigarette smoking and a depressive mood are highly co-morbid; however, the mechanism underlying this association is poorly understood [1]. Cigarette smokers often report that smoking can alleviate feelings of depression; thus feeling depressed may cause people to smoke as a way of self medicating. Alternatively, smoking may increase the risk of low mood via alterations in neurotransmitter pathways following chronic exposure. A further explanation is that other factors (confounders), such as social position, explain the observed association. Unfortunately observational studies cannot provide evidence of causation and so are unable to resolve this question [1].

Cigarette smoking during pregnancy is associated with increased risk of infant morbidity and mortality [2], [3], a fact which is made clear to women through concerted health education campaigns aimed at stopping women from smoking during pregnancy [4], [5]. Thus pregnancy is a situation characterized by considerable health and social pressure to stop smoking. Pregnancy therefore provides us with a window in which, rather than women stopping smoking for personal health reasons, there are women who stop smoking in high numbers because of concern for the health of their offspring. If smoking causes depressive mood, women who continue to smoke through pregnancy should report higher levels of depressed mood than those who stop smoking. However, if low mood rendered it difficult for women to give up smoking the same association would be seen. Similarly, if sociodemographic and behavioural factors were related both to the inability to stop smoking and to depressive mood ((i.e, the association is generated by confounding), the same association would again be seen. Therefore, in order to be able to make a casual interpretation in this situation we need an instrument (a surrogate for measuring smoking) which influences the tendency to smoke during pregnancy independently of mood.

Mendelian randomization uses genetic variation with known functional consequences to examine the causal effects of a modifiable exposure, such as tobacco use, on an associated outcome, such as depression [6]. In this study, we used a genetic variant known to be associated with continuing smoking during pregnancy, to estimate the effect of smoking on depressed mood. As genotype is determined at conception it cannot be susceptible to reverse causation (although it is theoretically possible that the same genotype could itself be associated with depressed mood). The rs1051730 genetic variant in the nicotine acetylcholine receptor gene cluster (*CHRNA5-CHRNA3-CHRNB4*) has been found to be robustly associated with number of cigarettes smoked per day among those who smoke in genome wide association studies [7], [8]. We have previously showed an association between this SNP and the risk of continuing smoking during pregnancy in a large population based study of pregnant women [9]. To the best of our knowledge, there have been no previous reports showing an association of this genotype with depression or depressed mood. We used this same cohort of women to determine whether continued smoking during pregnancy is associated with an increased risk of depressed mood, using rs1051730 as an instrumental variable in a Mendelian randomization analysis.

## Methods

### Sample

**Figure 1:**
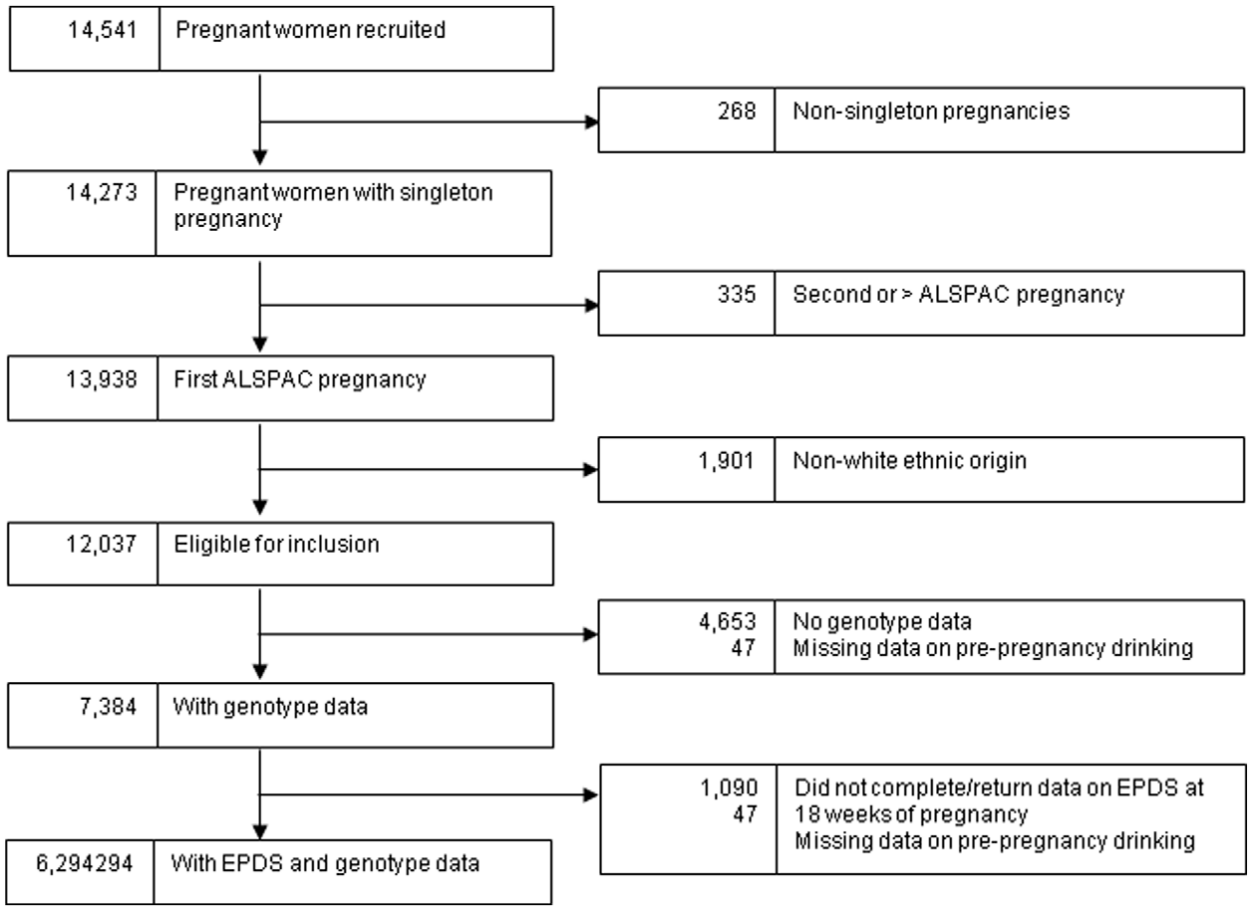
Flow-diagram of participants in the study, and reasons for exclusions.

The Avon Longitudinal Study of Parents and Children (ALSPAC) is a population-based prospective study investigating factors that affect the health and development of children and their parents. The study methods are described in detail elsewhere (http://www.alspac.bris.ac.uk and [10]). In brief, pregnant women living in Bristol, England who had an expected date of delivery between April 1991 and December 1992 were eligible to participate in the study. 14 541 pregnant women enrolled in the study. Detailed information on exclusion criteria for this analysis and numbers with missing data is given in Figure 1.

Ethical approval for the study was obtained from the ALSPAC Law and Ethics Committee and from three health authorities covering the Bristol area in November 1989 (Bristol and Weston), April 1990 (Southmead) and June 1990 (Frenchay). Written consent was obtained from all participants who took part in this study.

### Measurement of depressed mood

Women completed the Edinburgh Postnatal Depression Scale (EPDS) by questionnaire at 18 and 32 weeks of pregnancy. This questionnaire has 10 questions each with a maximum score of 3 points, with a higher score indicating a greater level of depressed mood. We used a cut-off point of greater than or equal to 13 on the EPDS to determine women with depressed mood for our primary analyses. This is a well-established cut-off point for determining low mood in pregnancy and in the postpartum period [11], [12]. Sensitivity analyses were also carried out using cut-offs of greater than or equal to 10, 11 and 12.

### Measurement of smoking status

Smoking behaviour of women before and during pregnancy was determined from self-reported questionnaires completed at 18 weeks gestation, asking about lifetime, pre-pregnancy and first trimester smoking status (current smoker, former smoker, never smoker) and, among current smokers, quantity of cigarettes smoked. Data on smoking quantity among current smokers was categorized into <10 and ≥10 cigarettes per day.

### Genotyping

Genotyping was undertaken by KBioscience Ltd. Ltd (www.kbioscience.co.uk), who use their own form of competitive allele specific PCR system (KASPar) and Taqman™ for SNP analysis.

### Statistical analysis

Logistic regression analyses were used to test for associations between variables with caseness based on the EPDS (score ≥13) as the outcome. The interaction between genotype and smoking on EPDS score at 18 weeks pregnancy was tested by comparing a model with and without an interaction term between the two variables, using a likelihood ratio test. If genotype is associated with depressed mood independently of smoking status we should see a similar association between genotype and depressed mood for both smokers and non-smokers pre-pregnancy, and no interaction between genotype and smoking status on risk of depressed mood. Assumptions of Hardy-Weinberg Equilibrium were formally tested using a chi-squared test. Instrumental variable estimation of the effect of continuing smoking on the risk of depressed mood (defined by an EPDS score of greater than 12) was performed using two-stage least squares using the rs1051730 genotypes under an additive model as the instrumental variable. Two stage least squares estimation proceeds by first fitting the regression of continuing smoking (exposure) on rs1051730 genotypes (instrument). In the second stage the depressed mood indicator (outcome) is regressed on the predicted values of continuing smoking, the coefficient of which is the estimate of the causal effect. Importantly the standard errors of the second stage parameter estimates are appropriately corrected (increased) to account for the uncertainty in the predicted values of the exposure from the first stage. All analysis was done using Stata 11.0 (Stata Corporation, College Station, Texas).

## Results

### Pre-pregnancy smoking status and depressed mood at 18 weeks of pregnancy

Of the 6294 women included in this study, 33.2% smoked prior to becoming pregnant, 26.8% of whom (560/2 090) reported quitting smoking in the first trimester of pregnancy.13.6% of women in this study reporting a depressed mood at 18 weeks of pregnancy (i.e., a score of 13 or more on the EPDS), with the women who reported smoking prior to pregnancy being 2.5 times more likely to report having a depressed mood at 18 weeks of pregnancy (OR = 2.47, 95% CI 2.13 to 2.86, p<1×10^−20^). Women who continued smoking during pregnancy reported slightly higher levels of depressed mood at 18 weeks pregnancy compared to those who stopped (OR = 1.21, 95% CI 0.94 to 1.53, p = 0.133).

### Genotype and pre-pregnancy smoking status

The T allele at the rs1051730 locus was present at a frequency of 33% in this study (prevalence of TT = 11.22%, CT = 44.22%, CC = 44.6%), and genotypes showed little evidence of deviation from Hardy Weinberg equilibrium (p = 0.60). We found that in the 6924 women with EPDS score data at 18 weeks of pregnancy, rs1051730 genotype was associated with an increased risk of continuing smoking during pregnancy (per allele effect for each T allele OR = 1.31, 95% CI 1.13 to 1.52, p = 0.0004), and with an increased risk of smoking ≥10 cigarettes a day pre-pregnancy (per allele effect for each T allele OR = 1.21, 95% CI 1.04 to 1.40, p = 0.013). This is in concordance with a previous report by Freathy and colleagues (10), who investigated smoking characteristics by genotype in a larger sample of women from the same cohort.

### Genotype and depression

Overall there was very little evidence of an association between genotype and depressed mood (EPDS≥13) at 18 weeks of pregnancy (per allele OR = 0.94, 95% CI 0.84 to 1.05, p = 0.25). Among women who smoked prior to pregnancy, however, the T allele was associated with a reduced risk of reporting a depressed mood (per allele OR = 0.84, 95% CI 0.72 to 0.99, p = 0.034). Moreover, the risk of depressed mood among TT homozygotes was almost half that of CC homozygotes (OR = 0.56, 95% CI 0.37 to 0.84, p = 0.005), with little evidence of an association between genotype and depressed mood among women who were non-smokers prior to pregnancy (per allele OR = 1.03, 95%CI = 0.86–1.20, p = 0.70). There was weak evidence of an interaction between genotype and smoking prior to pregnancy (p = 0.07) on the risk of depressed mood at 18 weeks of pregnancy. These results are presented in Table 1. Sensitivity analyses using EPDS score cut-offs of ≥10, ≥11, and ≥12 among smokers showed that a cut-off of ≥12 gave results very similar to those using ≥13, whereas using a less stringent EPDS cut-off to define the outcome gave results which were attenuated (results available on request). Using data at 32 weeks of pregnancy produce broadly similar findings. These results are presented in Table 2.

**Table 1:** Risk of depression at 18 weeks of pregnancy by rs1051730 genotype, stratified by pre-pregnancy smoking status.

EPDS> = 13	rs1051730 SNP			Total	OR (95% CI) p-value		
	CC	CT	TT		CT versus CC	TT versusCC	Per allele effect
*All women*							
Not depressed	2423 86.3%	238886.0%	62988.8%	544086.4%	1.03(0.88–1.20)P = 0.72	0.79(0.61–1.02)P = 0.07	0.94(0.84–1.05)P = 0.25
Depressed	38513.7%	39014.0%	7911.2%	85413.6%			
Total	2808	2778	708	6294			
*Smokers*							
Not depressed	72177.9%	72678.0%	20186.2%	164878.8%	0.99(0.80–1.24) p = 0.95	0.56(0.37–0.84) p = 0.005	0.84(0.72–0.99)P = 0.034
Depressed	20522.1%	20522.0%	3213.7%	44221.2%			
Total	926	931	233	2090			
*Nonsmokers*							
Not depressed	170290.4%	166290.0%	42890.1	379290.2	1.05(0.85–1.31) p = 0.64	1.04(0.74–1.46) p = 0.83	1.03(0.86–1.20)P = 0.70
Depressed	1809.6%	18510.0%	479.9%	4129.8%			
Total	1882	1847	475	4204			

P-value for interaction between pre-pregnancy smoking and genotype = 0.07 for per allele model.

**Table 2:** Risk of depression at 32 weeks of pregnancy by rs1051730 genotype, stratified by pre-pregnancy smoking status.

EPDS> = 13	rs1051730 SNP			Total	OR (95% CI) p-value		
	CC	CT	TT		CT versus CC	TT versusCC	Per allele effect
*All women*							
Not depressed	131483.8%	127183.8%	33284.0%	291783.8%	0.99(0.82–1.20)P = 0.95	0.98(0.72–1.32)P = 0.88	0.99(0.87–1.13)P = 0.89
Depressed	25516.2%	24516.2%	6316.0%	56316.2%			
Total	1569	1516	395	3480			
*Smokers*							
Not depressed	44884.4%	43583.5%	12088.9%	100384.5%	1.07(0.77–1.48) P = 0.70	0.67(0.38–1.21) P = 0.19	0.91(0.72–1.15)P = 0.43
Depressed	8315.6%	8616.5%	1511.1%	18415.5%			
Total	531	521	135	1187			
*Nonsmokers*							
Not depressed	86683.4%	83684.0%	21281.5%	191483.5%	0.96(0.76–1.21) P = 0.72	1.14(0.80–1.62) P = 0.47	1.03(0.88–1.22)P = 0.70
Depressed	17216.6%	15916.0%	48 18.5%	37916.5%			
Total	1038	995	260	2293			

P-value for interaction between pre-pregnancy smoking and genotype = 0.18 for per allele model.

Using rs1051730 genotype as an instrumental variable, the estimated causal effect of continuing smoking on depressed mood, on the risk difference scale, was −54.7% (95% CI −114.3% to 4.8%). This is consistent with a *decreased* risk of reporting a depressed mood rather than an increased risk, given continued smoking during pregnancy.

## Discussion

In this study, the rs1051730 T allele, which is associated with increased number of cigarettes smoked per day and reduced ability to stop smoking during pregnancy, appears to be associated with lower levels of reporting of depressed mood. This analysis does not provide evidence in support of the hypothesis that smoking causes depressed mood. Women with the T allele were more likely be heavier smokers prior to pregnancy, and to continue smoking in the first trimester of pregnancy when pressure to stop smoking was high, but were less likely to report a depressed mood at 18 weeks of pregnancy. Our results are consistent with the hypothesis that tobacco withdrawal causes symptoms of depressed mood, since the C allele was associated with stopping smoking and with an increased likelihood of reporting a depressed mood during pregnancy. However, in this study smoking cessation was associated with an overall reduction in the reporting of low mood among pregnant women. Taken together, our results support a self-medication explanation for the observed association between smoking and depressed mood symptoms.

Several limitations need to be borne in mind when considering these results. First, we did not conduct psychiatric interviews to assess depression, but instead used the EPDS [11] to assess depressed mood. This is a self-reported questionnaire, which has been shown to be a valid tool for measuring depression during and immediately following pregnancy (sensitivity 79%, specificity 85%) in the UK and elsewhere [13]. However, a high score on the EPDS does not constitute a diagnosis of depression. Second, once we had excluded all women with missing data (the main reason being lack of DNA) we were left with a much smaller subset of the original ALSPAC study. However, our depressed mood scores corresponded very well with those reported previously in a much larger sample [14], suggesting that the women included in this analysis were representative of the women who took part in the overall study. Third, whilst it is unlikely that this genetic variant exhibits effects on depressed mood that are independent of those on smoking, our findings do raise a potential issue of pleiotropy. This could be addressed in the future by using multiple independent genetic instruments, which influence smoking cessation independently via different pathways [15]. In our view, it is more likely that a synergistic relationship between genotype and smoking on mood exists, rather than an independent effect of genotype on depressed mood because there is no effect of genotype on depression among women who were non-smokers. Fourth, we did not measure other clinical outcomes related to depressed mood, such as anxiety. We therefore cannot say to what extent the relationships we observed reflect anxiety rather than a depressive tendency. However the co-morbidity of anxiety and depressed symptoms is so high that it may be difficult to find specific associations. Fifth, smoking status was assessed by self-report and without biochemical verification. The use of a genetic marker as a proxy for smoking status during pregnancy reduces the potential impact of this limitation, as this is unlikely to vary strongly as a function of reporting bias. Nevertheless, objective assessment of smoking status and cigarette consumption would be preferable in future studies.

A recent study showed that the minor allele of the missense polymorphism, D398N, in *CHRNA5* (rs16969968), which is highly correlated with the T allele of rs1051730 (r^2^ = 0.79), conferred a reduced response to a nicotinic agonist in vitro [16]. The α5 nicotinic acetylcholine receptor subunit encoded by this gene participates in multiple nicotinic receptor subtypes, including the α4β2α5 subtype that contributes to nicotine-stimulated dopamine release in the striatum. This region is involved in the reward pathway and is crucial to the development of substance dependence [17], [18], and may affect mood as well as smoking behaviour, given that high affinity nicotinic receptors in the brain can be desensitized by chronic nicotine use, leading to blunted cholinergic activity and a decrease in depressive symptoms. This has resulted in nicotinic antagonists recently being tested as treatments for depression in human subjects, particularly as adjunctive therapy together with classical antidepressants. Therefore, smokers may increase nicotine intake to avoid the aversive effects of withdrawal arising from lower plasma nicotine levels. Nicotine administration in this context can reduce symptoms of anxiety and depression, but nicotine also stimulates inhibitory 5-HT autoreceptors, which can itself be anxiogenic and/or depressogenic. Individuals with reduced nicotine-cholinergic receptor activity due to genetic variation may therefore require greater amounts of nicotine to achieve the same activation of the dopaminergic pathway.

Our data are not consistent with a causal effect of smoking on depressed mood, but while we cannot directly conclude from our data that depressed mood leads to smoking, this is one possible explanation for the results we observe. Women homozygous for the rs1051730 T allele, who were least likely to stop smoking during pregnancy, showed the lowest levels of depressed mood. This led to an instrumental variable estimate which suggests that continued smoking during pregnancy reduces the risk of depressed mood. We propose that there may be two factors determining whether women continue to smoke during pregnancy. One is tobacco dependence, which is at least partly influenced by rs1051730 genotype, and the other is depressed mood. These mechanisms appear to be largely independent of each other. This may serve to guide both the treatment of individuals who wish to stop smoking, and the extent to which tobacco use may be regarded as a coping strategy. For example, public health programmes aimed at helping women to stop smoking during pregnancy should adopt a two-pronged approach to address women's general wellbeing, including their mental health.
